# Conversas, interações e reações emocionais durante as visitas familiares ao QuitoZoo, Equador

**DOI:** 10.1590/S0104-59702025000100045

**Published:** 2025-10-13

**Authors:** Jéssica Beck Carneiro, Luisa Massarani, Graziele Scalfi, Martín Bustamante, Gabriela Arevalo, Jorge Heredia

**Affiliations:** i Pesquisadora bolsista, Instituto Nacional de Comunicação Pública da Ciência e Tecnologia. Rio de Janeiro – RJ – Brasil jessicabcarneiro.inct@gmail.com; ii Coordenadora, Instituto Nacional de Comunicação Pública da Ciência e Tecnologia;pesquisadora, Casa de Oswaldo Cruz/Fiocruz. Rio de Janeiro – RJ – Brasil luisa.massarani@fiocruz.br; iii Pesquisadora bolsista, Instituto Nacional de Comunicação Pública da Ciência e Tecnologia. Rio de Janeiro – RJ – Brasil graziscalfi@gmail.com; iv Diretor, Zoológico de Quito/Fundação Zoológica do Equador. Quito – EC – Equador mbustamante@quitozoo.org; v Diretora de educação para a conservação, Zoológico de Quito/Fundação Zoológica do Equador. Quito – EC – Equador gabrielaarevalog@gmail.com; vi Gestor de experiências, Zoológico de Quito/Fundação Zoológica do Equador. Quito – EC – Equador jorgeh@quitozoo.org

**Keywords:** Estudo de público, Educação ambiental, Biodiversidade, Audience research, Environmental education, Biodiversity

## Abstract

Este estudo qualitativo e exploratório visou aprofundar o conhecimento sobre a experiência familiar em zoológicos. Analisando conversas, interações e emoções, procuramos entender como os grupos familiares constroem estratégias e interpretam o significado da visita. Nove grupos familiares participaram das gravações no QuitoZoo, sendo o material carregado no software Dedoose e categorizado por um protocolo prévio, com posterior análise das categorias perceptivo, biológico e conexão. Os familiares interagiram e conversaram sobre temas de ciência que refletiam emoções e conexões. Usaram estratégias de envolvimento cognitivo e construíram significado a partir das experiências na exposição, o que pode auxiliar na aprendizagem e levar, a longo prazo, ao desenvolvimento de valores de conservação.

## Abstract (en)

This qualitative, exploratory study aimed to deepen knowledge about family experience at zoos. By analyzing conversations, interactions and emotions, we sought to understand how family groups build strategies and interpret the meaning of the visit. Nine family groups participated in the recordings at QuitoZoo. The material was uploaded to Dedoose software and categorized according to a pre-established protocol, followed by the analysis of the perceptual, biological, and connection categories. The family members interacted and discussed scientific topics that reflected emotions and connections. They used cognitive engagement strategies and constructed meanings from their experience at the exhibit, which can aid learning and lead, in the long term, to develop conservation values.

A visita familiar ao zoológico é um fenômeno multifacetado, influenciado por uma diversidade de fatores e motivações. Esses espaços são reconhecidos por sua missão de conservação das espécies e do patrimônio natural (Azab, dez. 2019; Skibins, Dunstan, Pahlow, 2017). Também se destacam no âmbito educacional ao enriquecer o conhecimento sobre a fauna e flora e promover o contato com a natureza e sua contemplação (Galheigo, Santos, 2009).

Para além dessas questões, os visitantes de zoológico associam a visita a um momento de diversão, exploração do mundo natural e lazer com a família e os amigos (Clayton, Fraser, Saunders, 2009). Ao vivenciar um dia agradável fora do ambiente convencional e se divertir, os genitores veem a experiência como uma oportunidade de fornecer informações interessantes às crianças (Morgan, Hodgkinson, 1999) e interagir com animais (Pekarik, 2004; Clayton, Fraser, Saunders, 2009).

Nesse sentido, os espaços não formais de educação se destacam por oportunizar o encontro dos visitantes, especialmente o das crianças urbanas, com animais vivos (Kisiel et al., 2012; Patrick, Tunnicliffe, 2013; Rowe, Kisiel, 2012). A presença física desses animais oferece uma vantagem significativa para a aprendizagem (Pearson, Dorrian, Litchfield, 2013). Além disso, permite uma vivência controlada em um ambiente que enfatiza transmitir conceitos sobre a conservação (Clayton, Fraser, Saunders, 2009).

Em geral, nesses espaços, são realizados estudos e ações relacionadas ao monitoramento de espécies ameaçadas de extinção e o resgate de animais silvestres do tráfico e da biopirataria (Marin, Carvalho, Freitas, 2017). Desenvolvem-se, também, técnicas para produção e manejo visando à reintegração das espécies em seu *habitat* natural (Iared, Tullio, Oliveira, 2012).

Nas últimas décadas verifica-se aumento no número de pesquisas que abordam os agrupamentos familiares e examinam como ocorrem as interações interpessoais entre pais e filhos em espaços não formais (Kisiel et al., 2012; Patrick, Tunnicliffe, 2013; Rowe, Kisiel, 2012). No entanto, poucos estudos sobre o envolvimento familiar com animais vivos se concentraram em analisar suas conversas, especialmente, como ocorre o desenvolvimento do conhecimento biológico sobre os animais (Geerdts, Walle, Lobue, 2015).

Geerdts, Walle, Lobue (2015) abordaram a temática familiar em zoológicos, concentrando-se em averiguar se os pais fornecem informações que apoiem o conhecimento biológico emergente das crianças nesse ambiente. Em seu estudo, utilizaram as categorias perceptivo, conceitual, biológico, social e conexão para classificar os tipos de conversas recorrentes em grupos familiares durante a visita. Para os autores, as interações familiares durante as visitas revelam oportunidades de aprendizagem que podem influenciar o desenvolvimento das primeiras teorias biológicas e psicológicas das crianças sobre os seres vivos (Geerdts, Walle, Lobue, 2015). Detalhamos a seguir as categorias perceptivo, biológico e conexão, as quais utilizamos como base para classificar as conversas mais representativas do presente estudo.

A categoria perceptivo refere-se a falas, interações com imagens ou gesticulações, como apontar e contar, ao identificar os animais, rotulando-os e atribuindo-lhes características físicas (Geerdts, Walle, Lobue, 2015). Como descrito em estudos prévios, o tempo de permanência ao visualizar (Luebke et al., 2016) e apontar (Bangerter, 2004) podem ajudar os visitantes a manter o foco e a atenção em ambientes estimulantes.

Para Povis e Crowley (2015), a atenção conjunta vai além da covisualização, necessitando atingir um envolvimento cognitivo compartilhado em torno de estímulos focais e assuntos específicos que encorajam as estratégias de exploração. Dessa maneira, funcionam como uma oportunidade de conversação sobre um mesmo referencial, permitindo mais tempo de processamento, aprofundamento e retenção de informações (Kim, Mundy, 2012; Povis, Crowley, 2015).

Entre as estratégias que podem auxiliar os visitantes a manter o foco estão também as placas expositivas. Muitos visitantes veem nas placas uma forma de adquirir conhecimentos que buscam sobre os animais (Aragão, 2014). Dessa forma, seu conteúdo informativo torna-se essencial para os visitantes que buscam identificar, aprimorar conhecimentos e satisfazer a curiosidade sobre os animais.

Patrick e Tunnicliffe (2013) trazem o conceito de “a voz do zoológico”, ao afirmar que recinto/animal vivo/placa expositiva é a principal unidade expositiva no zoológico. Para os autores, todo o ambiente deve ser pensado para despertar a curiosidade nos visitantes e, consequentemente, sensibilizar as pessoas para as questões ambientais.

Igualmente, revela-se essencial a qualidade dessas placas. A atratividade visual, argumentada por Saraiva (2017), desempenha papel significativo no despertar do interesse dos visitantes, ao combinar imagens envolventes e informações relevantes, enriquecendo a experiência sensorial e ampliando o engajamento cognitivo compartilhado (Aragão, 2014).

Além das placas expositivas, a história dos zoológicos está enraizada na exibição de animais que chamam a atenção do público, por conta de sua atratividade e de seu amplo apelo social (Skibins, Dunstan, Pahlow, 2017). Nesse sentido, suas características físicas e estéticas são determinantes, estando os mamíferos no topo da popularidade (Moss, Esson, 2010; Small, 2012).

A relação entre a atratividade dos animais e a aprendizagem potencial dos visitantes é uma constante nos zoológicos, sustentando-se nas sugestões de Moss e Esson (2010), que examinaram esse interesse relativo e a atração dos visitantes em quarenta zoológicos. As espécies emblemáticas demonstraram ter o poder de impulsionar ações em prol da conservação, reforçando uma conexão que remonta à história dos zoológicos, conforme apontado por Martin et al. (2014). Apesar dessa atratividade inicial, de forma geral, todos despertam algum tipo de curiosidade e estimulam o envolvimento cognitivo dos visitantes (Clayton, Fraser, Saunders, 2009), como será descrito na próxima categoria.

A categoria biológico se refere às falas sobre processos e informações biológicas dos animais, como dieta alimentar, ciclo de vida, aspectos relacionados à conservação e riscos de extinção, tráfico, entre outros. Em zoológicos, espaços propícios para o diálogo sobre temas relacionados aos animais e a seus processos biológicos, as conversas são consideradas um importante mecanismo para a aprendizagem (Kim, Mundy, 2012; Povis, Crowley, 2015). A forma como são conduzidas e o seu conteúdo podem impactar o modo como as crianças raciocinam sobre os tipos de animais (Geerdts, Walle, Lobue, 2015).

As famílias se envolvem na investigação científica ao articular seus pensamentos e manifestar seus raciocínios científicos durante suas conversas nesses espaços (Leinhardt, Crowley, Knutson, 2002; Povis, Crowley, 2015). Tal envolvimento cognitivo e de raciocínio é expresso em perguntas, hipóteses, evidências para comunicar seus pensamentos e também ao revisar teorias sobre as temáticas expostas (Bell et al., 2009; Crowley et al., 2001; Kisiel et al., 2012; Land-Zandstra, Hoefakker, Damsma, 2020).

A linguagem utilizada, as experiências prévias e as comparações são essenciais para o desenvolvimento desse raciocínio, durante a interação social, ao expor pensamentos cotidianos, envolvendo causalidade e contextualização (Massarani et al., 2022). Bell et al. (2009) comentam esses conceitos ao discutir as formas de aprendizagem entre pais e filhos sobre ciência e argumentam que associar o pensamento científico a eventos envolventes relacionados ao cotidiano pode criar conexões em níveis pessoal e coletivo, além de ser uma estratégia de aproximação à exposição (Silva, Gomes, 2009; Soares, Vieira, Fonseca, 2013).

É notável que, ao abordar temas biológicos, os familiares combinam raciocínio científico com abordagens mais intuitivas e explicativas, incorporando concepções alternativas. Essa mesclagem simplifica o conteúdo científico, facilita a expressão dos pensamentos e promove uma aprendizagem mais acessível. Além de servir como via de transição conceitual entre as formas de raciocínio (Massarani et al., 2022).

Assim, os familiares coconstroem o pensamento científico (Crowley et al., 2001). Por isso, o envolvimento em conversas explicativas pode ser mais uma forma pela qual os pais promovem a aprendizagem biológica nesses espaços (Legare, Lombrozo, 2014; Geerdts, Walle, Lobue, 2015). Essas explicações podem contribuir também para o aprofundamento de teorias e estimular as habilidades de resolução de problemas científicos (Fender, Crowley, 2007).

Também, a resposta reflexiva revela um nível adicional de processamento cognitivo ao provocar o significado pessoal da experiência (Ballantyne, Packer, Falk, 2011). Isso evidencia que os visitantes usam o zoológico para construir sentido, a partir das experiências deles, o que impulsiona a compreensão pessoal, significativa para eles ou para seu grupo, como visto em pesquisas museais, como a de Falk e Dierking (2000).

As temáticas animais e os seus processos biológicos ganham destaque nesses ambientes. Fraser et al. (2009) mencionam que a dieta alimentar foi um dos assuntos mais procurados pelos visitantes de cinco instituições norte-americanas. Além de refletir tais hábitos, esse tema se conecta diretamente às experiências dos visitantes, sendo um tópico recorrente nas conversas. Já o cuidado parental foi identificado em Carr (2016) como uma das características consideradas mais atrativas em um zoológico.

Skibins, Dunstan e Pahlow (2017) identificaram que o estado de conservação de uma espécie influenciou a conexão emocional e as intenções comportamentais dos visitantes. Esse resultado é importante, na medida em que a construção de zoológicos como centros de reprodução para animais ameaçados de extinção pode se encaixar na conceituação do público sobre características desejáveis em animais nessas instituições. Também pode auxiliar essas instituições a atingir sua meta sobre a conservação por meio da educação dos visitantes em proteger a vida selvagem (Clayton, Fraser, Saunders, 2009).

Independentemente do tema biológico, a alimentação, o cuidado parental, a camuflagem, a conservação, a extinção e o tráfico emergem como focos centrais das conversas e enfatizam sua relevância na interação dos visitantes com zoológicos (Fraser et al., 2009). A categoria conexão faz referência às falas comparativas entre um animal e as suas características com outro animal ou objeto, relacionam os animais ou o zoológico às experiências anteriores e esboçam emoções (Geerdts, Walle, Lobue, 2015).

Além dessa combinação de informações, o processo de aprendizagem está sob controle direto do interesse e das experiências anteriores do indivíduo, fatores fundamentais na percepção, reação e, em última análise, aprendizagem nesses ambientes (Heimlich, Horr, 2010). Namy e Gentner (2002) assinalaram que, ao conversar, adultos e crianças aproximaram o conteúdo expositivo das vivências prévias (Silva, Gomes, 2009; Soares, Vieira, Fonseca, 2013), estratégia que, por si só, o torna mais compreensível (Leinhardt, Crowley, Knutson, 2002).

As emoções são um conjunto de componentes psicológicos e físicos que ocorrem simultaneamente em resposta a experiências cognitivas, fisiológicas, comportamentais e afetivas, como os sentimentos (Scherer, Moors, 2019). Nesse sentido, esboçar emoções também pode ser uma forma de potencializar o processo de retenção, compreensão e transferência do conhecimento (Caine, Caine, 1994), principalmente quando os visitantes manifestam emoções pelos animais e pela natureza (Rupp, 1999). As emoções positivas, aqui caracterizadas como a percepção sobre algo que gera aumento de energia e que envolve sentimentos de excitação, alegria e entusiasmo, levam a um envolvimento inicial familiar com a exposição com efeito facilitador das experiências acima citadas (Ballantyne, Packer, Falk, 2011; Luebke et al., 2016).

Allen et al. (2002) e Clayton, Fraser, Saunders (2009) sugerem que a resposta emocionalmente positiva a um animal está associada ao apoio a iniciativas de conservação. Carter (2011) argumentou que as emoções, como admiração, levam as pessoas a desempenhar comportamentos ambientalmente responsáveis. Nesse sentido, os zoológicos podem funcionar como espaços de valorização e cuidado dos animais e do ecossistema onde vivem, podendo desenvolver práticas e comportamentos pró-ambientais (Luebke et al., 2016).

As emoções negativas, aqui caracterizadas como a percepção sobre algo que gera desconforto e envolve sentimentos de aflição, agonia e antipatia, nos zoológicos, geralmente estão associadas a animais muito diferentes dos humanos que acionam emoções como medo. Estudos demonstram que os répteis e os insetos são mais suscetíveis a despertar tais emoções (Skibins, Dunstan, Pahlow, 2017). Santos et al. (2020) também demonstram emoções negativas (decepção e impaciência), quando os visitantes tentam interação próxima com os animais, mas não conseguem.

Apesar disso, em estudos recentes, as emoções negativas desempenham um papel significativo, sugerindo um fascínio que motiva a observação desses animais, conduzindo a mais atenção e até mesmo a comentários positivos, além de contribuir para a conexão com animais (Pekarik, 2004). São igualmente importantes para as experiências das crianças, podendo estimular maior concentração e engajamento (Rappolt-Schlichtmann, 2017).

Dessa forma, o objetivo deste estudo qualitativo e exploratório foi aprofundar o conhecimento sobre a experiência familiar em zoológicos e entender como os familiares constroem e interpretam o significado situacional da visita, analisando suas conversas, interações e emoções.

## História do QuitoZoo

O Zoológico de Quito (QuitoZoo) funciona há 27 anos em Guayllabamba, uma freguesia rural do distrito metropolitano de Quito. Esse imóvel municipal de 12 hectares foi construído para receber a população animal do antigo Zoológico Amazonas da Escola Militar Eloy Alfaro, quando fechou suas portas e seu terreno foi entregue para a construção de um hotel cinco estrelas. Desde o encerramento, a prefeitura de Quito assumiu a função de administração municipal, apoiando essa instituição.

Após dois anos de gestão, em 2000, a prefeitura de Quito entregou o zoológico para ser administrado por um contrato de empréstimo da Fundação Zoológica do Equador, uma organização privada não lucrativa, formada em 1994. Atualmente o QuitoZoo funciona como um centro de resgate do tráfico de animais silvestres e animais de estimação. Abriga milhares de histórias de seres selvagens que foram reintroduzidos e outros que não conseguiram retornar ao seu *habitat* porque as circunstâncias em que chegaram os obrigaram a permanecer sob cuidados humanos. Nesse espaço recebem cuidados veterinários e os cuidados diários de que necessitam. O QuitoZoo também funciona como uma plataforma de educação ambiental e formação cidadã promovendo a reconexão das famílias com a natureza, garantindo o cuidado da vida animal e a mudança comportamental e os hábitos pró-ambientais.

## Missão, espaços de imersão e exposições educativas

O QuitoZoo segue os princípios estabelecidos na missão da fundação, que trata da “conservação da biodiversidade por meio do resgate e cuidado da vida selvagem e da formação de cidadãos e organizações públicas e privadas” (QuitoZoo, 16 fev. 2005). A ênfase da fundação está no cuidado da vida selvagem, voltada para espécies nativas equatorianas, e a exposição é baseada em espécimes resgatados do tráfico, embora possam expor espécies exóticas provenientes do tráfico de vida selvagem.

O QuitoZoo, por intermédio das equipes técnicas, faz parte dos esforços para a conservação de espécies ameaçadas e participa ativamente da implementação de planos de ação para a conservação de animais, como condor, urso andino, onça-pintada e primatas.

Recebe aproximadamente duzentos mil visitantes por ano. O público é majoritariamente familiar e chega nos finais de semana. Os meses mais movimentados são fevereiro e agosto.

O zoológico está localizado dentro de um remanescente de floresta seca interandina. O passeio é realizado por um caminho autoguiado que percorre 34 recintos de animais sem classificação específica, exceto a área de felinos e primatas. No exterior de cada recinto, existem placas informativas sobre a espécie e o seu histórico de resgate. Nos espaços de imersão aviária e casa noturna, o visitante pode ter uma experiência mais próxima com pássaros e corujas.

Existem três estações de interpretação ambiental: condores, ursos andinos e onças (espécies prioritárias devido ao estado de conservação), das quais apenas a estação de condores é um espaço fixo. Além disso, há áreas de interpretação interativa relacionadas à biodiversidade nativa: “O mundo dos insetos” e “Animais perto de mim”.

Em 2021, como parte da proposta de renovação de experiências no zoológico, foi intervencionada a exposição de sapos marsupiais (espécie típica de Quito e seus arredores) para atualizar o zoológico com uma proposta museográfica que combina elementos de natureza, paisagismo e jardinagem, exposição de artes visuais, escultura e terrários com animais vivos. Nessa exposição foi desenvolvido um programa educativo durante a visita. Além disso, no final daquele mesmo ano, foi reativado um borboletário de imersão em uma área de floresta tropical na qual podem ser observadas 12 espécies de borboletas representadas por cerca de quinhentos indivíduos.

## Coleta de dados

A coleta ocorreu de 7 a 17 de abril de 2022 e foi realizada por pesquisadores do QuitoZoo. As famílias foram abordadas na entrada do zoológico pelos pesquisadores que explicaram a pesquisa e deram instruções necessárias para a participação. As nove famílias abordadas demonstraram interesse em participar. Um dos responsáveis pela família preencheu o Termo de Consentimento Livre e Esclarecido (TCLE) com informações sobre os procedimentos e objetivos de pesquisa.

Na primeira etapa do estudo, o adulto responsável respondeu a um questionário com o objetivo de traçar o perfil socioeconômico dos visitantes. A segunda etapa consistiu na visita familiar ao QuitoZoo, realizada de forma livre e com mediadores podendo ser acessados quando necessário, em locais aleatórios ou previamente determinados no zoológico. Nessa etapa, uma criança usou na cabeça uma câmera GoPro durante toda a visita. A gravação registrava dados audiovisuais da família, com a adaptação do método *point-of-view camera*, usado em diversos estudos na área de ciências sociais (Lahlou, 2011; Glãveanu; Lahlou, 2012) e em estudos anteriores desse grupo de pesquisa (Massarani et al., 2019a, 2019b, 2019c, 2019d; Scalfi et al., 2022, 2023). Por fim, ao concluir a visita, os familiares entregavam a câmera aos pesquisadores.

## Sujeitos do estudo

Um grupo era considerado uma família quando seus membros eram biologicamente relacionados ou quando considerados pelos seus próprios membros (Briseño-Garzón; Anderson, 2012), sendo necessária alguma relação de responsabilidade pela criança. Cada grupo deveria ser composto por no mínimo um adulto e uma criança de até 12 anos completos. Para facilitar as análises das conversas e interações, foi estipulado número máximo de seis participantes.

Quarenta visitantes participaram da pesquisa, sendo 19 do gênero feminino (13 adultas e 6 crianças) e 21 do gênero masculino (13 adultos e 8 crianças). Em sua maioria, os adultos responsáveis eram os progenitores, à exceção de G8, na qual o tio era o responsável. O Quadro 1 sintetiza as informações sobre as famílias e o tempo de visita de cada uma.

**Quadro 1:** : Informações dos visitantes de cada grupo familiar e seu tempo de visita

Grupos	Local de residência	Membros	Gênero, parentesco, idade, ID	Tempo de visita
G1	La Troncal/Cañar	6	2♀ (mãe e avó); (36; 60); (A2; A4) 4♂ (pai, filhos e avô); (35; 8; 58; ?); (A1; C1; A3; C2)	1h12min18s
G2	Manta	4	3♀ (mãe, avó e filha); (39; 67; 7); (A2; A3; C1) 1♀ (pai); (44); (A1)	1h38min40s
G3	Quito	5	2♀ (mãe e filha); (45; 6); (A2; C1) 3♂ (pai e filhos); (47; 24; 14); (A1; A3; A4)	52min39s
G4	Manta	4	2♀ (mãe e avó); (36; 66); (A2; A3) 2♂ (pai e filho); (41; ?); (A1; C1)	1h3min19s
G5	Cotacachi	3	1♀ (mãe); (29); (A2) 2♂ (pai e filho); (31; 7); (A1; C1)	1h46min8s
G6	Calderón	4	3♀ (mãe e filhas); (30; 9; 3); (A2; C1; C2) 1♂ (pai); (38); (A1)	23min
G7	Portoviejo	6	4♀ (mãe, tia e filhas); (27; 54; ?; 2); (A2; A4; C1; C2) 2♂ (pai e tio) (35; 35); (A1; A3)	2h48min25s
G8	Machala	3	3♂ (tio e sobrinhos) (51; 11; 6); (A1; C2; C1)	1h46min15s
G9	Quito	5	2♀ (mãe e tia); (32; 22); (A2; A4) 3♂ (pai, tio e filho); (37; 25; ?); (A1; A3; C1)	1h4min39s
Total	-	40	19♀ e 21♂	12h35min23s

Fonte: elaboração dos autores (2024).

## Protocolo de análise dos dados

Para a análise das visitas, foi utilizado o protocolo desenvolvido pela rede de pesquisadores envolvidos no projeto, adaptado de Allard e Boucher (1998), que investigaram, nesse trabalho, as relações entre os atores fundamentais em uma exposição: módulos expositivos, mediadores e visitantes. O protocolo de análise e codificação das visitas está dividido em cinco dimensões, a saber: conversação; interação; emoção; fotos e mudança. Neste trabalho exploramos as de maior ocorrência, que seguiram para uma análise mais aprofundada: conversação, interação e emoção. No Quadro 2 apresentamos a descrição dessas categorias e subcategorias em suas respectivas dimensões.

**Quadro 2:** : Dimensões das categorias de análise de maior ocorrência nas visitas


1. CONVERSAÇÃO	
Conteúdo das conversações – Diálogos entre os visitantes estimulados pela interação com os módulos expositivos e/ou com os outros visitantes.	
1.1. Conversas sobre ciência	Diálogos sobre algum tema científico, discute dilemas éticos e morais da ciência, impacto social da atividade científica, trazem dados ou conteúdos científicos etc.
1.2. Conversas sobre animais	Diálogos envolvendo o reconhecimento de animais, fala sobre os animais e seus hábitos.
**2. TIPOS DE INTERAÇÃO**	
**Interação com o módulo expositivo –** Participação em módulos interativos, brincadeiras, vídeos, leitura e contemplação.	
**Interação visitante-módulo expositivo**	
2.1. Leitura de texto expositivo	A interação se dá pela leitura em voz alta de textos (integrais ou parte) de placas informativas, painel, legenda, texto, *charge*, dos módulos expositivos.
2.2. Interação contemplativa	Contemplação, observação, visualização sem toque/manipulação de um módulo expositivo ou parte específica dele.
**3. EMOÇÃO** Valoração afetiva com verbalizações de aspectos afetivos, emoções e sentimentos provocados pela exposição (medo, nojo, repulsa, desejo, admiração, espanto, surpresa, desconforto etc.).	

Fonte: elaboração dos autores (2024).

Para análise das visitas, fizemos uso do programa de análise qualiquantitativa, Dedoose 8.0.54, que categoriza segmentos de áudio e vídeo das ações corporais, textuais e das atitudes dos visitantes de maneira simultânea, otimizando a codificação. No programa, foram identificados os trechos das conversas, interações e respostas emocionais dos familiares, sendo aplicados códigos a cada uma dessas dimensões. É importante destacar que um código pode ocorrer em sobreposição a outro, visto que as categorias do protocolo de análise não são excludentes e podem acontecer simultaneamente em uma visita.

A partir das categorias e subcategorias mais representativas do mencionado protocolo de análise (Quadro 2), adaptamos ao nosso artigo as categorias perceptivo, biológico e conexão de Geerdts, Walle, Lobue (2015), que também não são autoexcludentes, analisando de forma qualitativa os resultados obtidos e classificando-os conforme o Quadro 3.

**Quadro 3:** : Classificação das categorias de análise mais representativas

Divisão	Descrição e exemplos
Perceptivo	Falas ou interações referentes às imagens visuais ou gesticulações, como apontar ou contar, que têm como objetivo direcionar a atenção do grupo familiar aos animais ou a algo exposto no zoológico. Exemplos: Chamar a atenção (“Olha!”); Identificação/rotulação (“Esses são macacos”); Atribuição de características físicas (“É grandona a avestruz”).
Biológico	Falas sobre processos e informações biológicas dos animais. Exemplos: Alimentação (“Come carne”); Cuidado parental (“O macho cuida do bebê”); Estratégias de defesa (“Está camuflada”); Ciclo de vida (“Há borboletas que passam mais tempo no casulo do que como borboletas?”); Conservação e extinção (“Vai se extinguir”); Tráfico de animais (“Você sabe o que é tráfico, né?”).
Conexão	Falas comparativas entre animais ou suas características que relacionam os animais ou o zoológico às experiências anteriores. Exemplos: Comparações com outro animal (“Parece uma rena”) ou objeto (“Eu pensei que era uma estátua”); Experiências anteriores (“São vegetarianos, nos disseram na escola”); Reações emocionais (“Que medo!”; “Que bonitos são os macacos!”).

Fonte: elaboração dos autores (2024), adaptado de Geerdts, Walle, Lobue (2015).

## Resultados

Os vídeos com as visitas familiares somaram 12h35min23s, sendo o tempo médio de visita de cada grupo 84 minutos. Os dados das interações dos grupos familiares, durante a visita ao QuitoZoo, por sua vez, contabilizaram 40h7min24s de vídeos analisados.

Conforme indicado, identificamos, por meio do uso do *software* Dedoose, as conversas e ações mais relevantes durante as visitas, sendo elas: conversas sobre ciência (n = 288; 12% de ocorrências), conversas sobre animais (n = 415; 17% de ocorrências), leitura de texto expositivo (n = 192; 8% de ocorrências), interação contemplativa (n = 225; 9% de ocorrências) e emoção (n = 332; 13% de ocorrências).

Para exemplificar os códigos utilizados nas análises foram usadas as letras “G” para se referir ao grupo, “A” ao adulto e “C” a criança. Os visitantes foram numerados de 1 a 6 em cada grupo, sendo o A1 o primeiro adulto a falar na gravação, A2, o outro participante adulto, e assim por diante, C1 a criança que está com a câmera acoplada na cabeça e C2 a outra. Por fim, analisamos qualitativamente e classificamos as conversas, interações e reações emocionais mais representativas, conforme as categorias adaptadas de Geerdts, Walle e Lobue (2015).

Os zoológicos, geralmente, são espaços de contemplação das espécies exibidas, e nesses espaços é um constante desafio manter o grupo atento ao que está observando. Assim, na categoria perceptivo, os grupos familiares utilizavam estratégias para manter ou direcionar a atenção aos animais ou à parte do zoológico que estavam observando no momento. Tais estratégias englobaram recursos visuais – com pronomes demonstrativos “Lá em cima”; interrogativos “E esse?”; e com o uso de verbo “Olha!” – juntamente ou não com o uso de gestos, como apontar ou contar (Ex. 1 e Ex. 2). Os familiares também mostraram repetidamente os animais ou as partes do zoológico aos demais familiares de forma que todos reconhecessem e se envolvessem naquele assunto, como pode ser visto a seguir:

Ex. 1. (G1): A2: E esse? C1: Essa é uma águia andina, já disseram. ... E de que cor é? C1: Vamos ver, não o vi, mas ali [no painel] leio que é preto e marrom. A2: Sim, muito bom. C1: Acho que só tem um!

Ex. 2. (G6): C1: Macaco! A2: Onde está? C1: Lá em cima na árvore. A2: Lá em cima. Olha, tem outro. ... A1: Olha! A2: Ah! Há outro, três. Um, dois, três, quatro.

Outra prática utilizada no zoológico foi rotular ou identificar os animais exibidos, atribuindo-lhes nomes populares ou científicos (Ex. 3 e Ex. 4), por vezes consultando as placas expositivas para identificação ou confirmação.

Ex. 3. (G3): C1: Olha, mamãe. Eu vi um macaco lá. Olha que lindo. A2: Esses são os macacos. A1: ‘Cabeça de mate’ [depois de ler silenciosamente].

Ex. 4. (G7): C1: Ai, meu Deus! Estes são maiores. [leitura] ‘*Cappu*…’ Não entendo. A1: ‘*Cappuccino*’. C1: *Cappuccino*, e ali botaram macaco. A1: Olha, aí está. C1: Eu vi. A2: Olha, naquele tronco ali. C1: Ali tem seis.

Para além de descobrir o nome dos espécimes, as placas expositivas foram utilizadas pelos grupos familiares que buscavam informações e curiosidades sobre os animais (Ex. 5 e Ex. 6) e, também, para a identificação por meio de uma fotografia ou ilustração (Ex. 1).

Ex. 5. (G1): C1: O avestruz! Olha como é grande! O avestruz é grande! ‘E botam uns maiores que outros’ [em relação aos ovos] [leitura].

Ex. 6. (G2): A2: E aqui é onde está a onça. A1: Olha, uma onça deitada aqui. A2 e A3: Ah, que linda! A1: Ele está dormindo. A1: É uma fêmea. C1: Olha a boca, quando ele acorda. [leitura] ‘A língua áspera serve como lixa para limpar a carne dos ossos’. Uau!

Ainda conversaram bastante sobre as características físicas dos animais, distinguindo-os entre machos e fêmeas (Ex. 7), conversando sobre sua morfologia (Ex. 8), coloração/pelagem e tamanho (Ex. 1).

Ex. 7. (G2): A3: Que lindo! C1: Sim, rica vida! ... A2: Isso é o quê? A3: Fêmea, é a fêmea. O macho tem pelagem e juba. C1: Sim. Eu sei o que é fêmea e o que é macho, a fêmea é quem não tem juba e o macho é quem tem juba.

Ex. 8. (G8): A1: Você viu? Como eles têm orelhas pontudas, né? C1: Sim.

O local de estudo também foi propício para diálogos sobre assuntos científicos e sobre animais. Logo, a categoria biológico se mostrou representativa com conversas sobre a alimentação, quando os familiares observaram os animais presentes no zoológico e fizeram inferências sobre a dieta alimentar do condor (Ex. 9) e da onça (Ex. 10).

Ex. 9. (G2): A2: Mas ele é carniceiro. A1: Carniceiro? A3: Sim, carniceiro, eles comem animais mortos.

Ex. 10. (G6): C1: Olha, e ali está a onça. Que bonita. E elas comem carne, então são selvagens.

Os familiares se envolveram na investigação científica e articularam o pensamento em conversas com os demais membros familiares e com mediadores. Embora tenham exibido um raciocínio mais científico, pautado na leitura de placas e nas explicações dos adultos, muitas vezes um viés mais científico ocorreu de forma mais intuitiva e explicativa, como no Ex. 11, que menciona o cuidado parental em leões bebês, e no Ex. 12, ao relacionar a espécie escondida à camuflagem.

Ex. 11. (G2): A2 e A3: O macho é quem cuida do bebê. C1: Olha, tem um aí. A3: Olha, eles são fiéis! ‘Eles escolhem um único parceiro e vivem juntos para o resto da vida” [leitura].

Ex. 12. (G7): C1: Aí está a ‘Indiana’! ‘Andina’! Mas eu não a vejo. A2: Olhe pra cima. C1: Já vi, é enorme, está camuflada. Está naquele galho alto, naquele galho ali. A2: Pronto, camuflada. Eu não a vi. É chamada de águia andina. O que diz aí? ‘São eles que têm penas nas patas’ [leitura]. C1: É ela. Já vi. Está naquele tronco longo.

Nessa categoria, não só os mediadores auxiliaram com informações científicas, como também os próprios familiares. Ambos os lados interagiram e se envolveram em conversas explicativas, questionamentos críticos e científicos, como no Ex. 13, quando os mediadores estimulam o raciocínio científico relacionado à conservação de espécies e à importância da polinização. Também quando falam sobre cuidado parental e ciclo de vida em sapos usando desenhos das placas informativas como referência, como no Ex. 14.

Nos exemplos a seguir (Ex. 13 e Ex. 14), também percebemos o uso de respostas reflexivas dos adultos, tanto familiares quanto mediadores, como forma de fazer os demais visitantes pensar reflexivamente sobre o assunto após uma explicação.

Ex. 13. (G2): M: E por que você acha importante ajudar a conservar essas aves? Você, o que acha? Por que os pássaros são tão importantes? A3: Porque ajudam a espalhar as sementes, a polinizar. M: Exatamente.

Ex. 14. (G3): A2: Os sapos, olha! C1: Eles são reais? A1: Eles são reais ou são estátuas? A1: A fêmea pode estar aqui. M: O que elas fazem é botar ovos nas costas, como você vê no desenho ali. E, quando estão prontas, entram na água e aqui estão. A1: Por que eles nascem tão pequenos? M: E aqui eles se transformam em sapinhos. E o que eles precisam para ser como os sapinhos daqui? Qual é a diferença? O que está faltando? A1: As patinhas. M: Sim, as patinhas.

Nas conversas sobre tráfico, conservação e extinção de espécies, o adulto exerceu o protagonismo ao utilizar as placas expositivas para comprovar informações ou reformular o conteúdo lido e explicá-lo de forma mais simples para a criança. É interessante destacar que os adultos leram nas placas que muitos dos animais do QuitoZoo foram resgatados dessa prática ilegal (Ex. 15).

Ex. 15. (G8): A1: Esse aqui se chama macaco “Ardilla Humboldt” [leitura]. C1: Eu gostaria de fazer carinho neles. A1: ‘Os macacos que chegaram ao zoológico foram entregues voluntariamente por pessoas que os tinham como animais de estimação. ... Essa espécie é conhecida pelo tráfico’. Você sabe o que significa tráfico, certo? C1: Sim. A1: Que as pessoas os tiram de onde viviam naturalmente e levam para vender. E há pessoas que os compram. ... C1: O macaco esquilo. A1 lendo: ‘O animal que você vê aqui é uma fêmea. Ela foi resgatada pela Unidade de Proteção Ambiental e pela Polícia Nacional. Em 2016, eles a mantiveram detida como animal de estimação’. Era como se ela fosse um cachorrinho.

Na categoria conexão, os grupos familiares fizeram comparações e associações com os animais exibidos com objetos (Ex. 16) e com outros animais (Ex. 17).

Ex. 16. (G1): C1: O condor? Esse é o condor? Onde está o condor? A1: Você já leu? C1: Sim, é um ‘condor andino’. A1: Ali! C1: Ah sim, pensei que fosse uma estátua.

Ex. 17. (G9): A1: Estes são leões africanos [depois de ler]. ... A4: São enormes, pensei que fossem mais ou menos do tamanho da onça. A1: Não, estes são maiores. Essa é uma leoa, ela já está velha, mas olha que fofa ela é. A4: Sim, o leão é mais velho.

Por vezes, referenciavam experiências anteriores e conhecimentos prévios com situações vividas na escola (Ex. 18) e com associações a filmes que já assistiram (Ex. 19).

Ex. 18. (G1): C1: Mais parecido com um urso andino, nos contaram na escola. A1: *National Geographic*. C1: Dizem que são vegetarianos, disse a professora. A1: Eles são veganos. A2: Muito bem.

Ex. 19. (G8): C2: Ah, condor! C1: Condor, onde eles estão? ... Eles vivem nas montanhas. C1: Olha, viu! C2: A cor equatoriana. Como no filme *Rio*. A1: Que cor linda!

Ainda nesse segmento, é interessante destacar que duas famílias relataram experiências anteriores no próprio QuitoZoo, como nos Ex. 20 e Ex. 21.

Ex. 20. (G6): C1: Come carne. A1: Claro! C1: Você lembra que da outra vez tinham dois aqui porque tinham pedaços de carne. ...

Ex. 21. (G8): A1: Tem algumas coisas que não lembro! Acho que o urso de óculos morreu. Então vou perguntar a alguém. C2: Olho de óculos.

A quase todo instante, os grupos familiares esboçaram emoções relacionadas ao ambiente e aos animais exibidos. Essas reações emocionais geralmente foram expressas em frases curtas que exibiam as respostas emocionais dos grupos familiares classificadas como positivas (encantamento, alegria e surpresa – Ex. 22) e negativas (medo e nojo – Ex. 23).

Ex. 22. (G6): C1: Ah, olha! Ah, olha, aí está, que lindo! Chama-se ‘macaco esquilo’. A1: Ah, que coisinha. C1: Ah, meu amor, meu amor!

Ex. 23. (G7): A2: Não pode tocar nas borboletas. A1: Sim, as asas não se tocam, mas, sim. C1: Uma vez uma parou no seu pé e te deu nojo. A1: Ela era muito grande e me deu medo, porque tinha cara de coruja*.*

## Discussão

Percebemos que os familiares foram receptivos às oportunidades de aprendizagem proporcionadas pelo zoológico, sendo motivados a visitá-lo, tanto pelo desejo de se divertir com a família quanto por conhecimento e educação, ao ver na visita uma oportunidade de fornecer informações interessantes aos seus filhos (Morgan, Hodgkinson, 1999).

Acreditamos que se conectar conscientemente à natureza já funciona como um produto do conhecimento e da aprendizagem familiar (Pearson, Dorrian, Litchfield, 2013). Especialmente para crianças urbanas com poucas oportunidades de encontrar animais vivos, zoológicos e museus desempenham um papel vital na exploração do mundo natural (Patrick, Tunnicliffe, 2013; Rowe, Kisiel, 2012), como visto nos trechos “(G1): A2: Você nunca tinha visto eles de verdade, né?” e “(G2): C1: Estou gostando muito desse zoológico. A4: Sim, ver animais que nunca pensei que veria pessoalmente. Digo, vivos!”.

Algumas estratégias de envolvimento e aprendizagem foram utilizadas pelos grupos familiares durante a visita. Na categoria perceptivo, emergiu a importância da atenção conjunta e a habilidade dos familiares de manter o foco em conversas e questionamentos substanciais e envolventes sobre os animais ou sobre as situações que vivenciaram. Estratégias simples, como apontar (Bangerter, 2004), aumentar o tempo de permanência durante a visualização conjunta (Luebke et al., 2016) ou promover diálogos sobre animais específicos, de forma que as famílias sustentassem conversas biológicas mais profundas, demonstraram ser eficazes para apoiar a aprendizagem (Kim, Mundy, 2012; Povis, Crowley, 2015).

Os zoológicos também funcionam como espaços em que os visitantes buscam por informações, seja o nome daquele espécime que estão vendo, seja uma informação para complementar seu conhecimento ou alguma curiosidade. Em Aragão (2014), alguns visitantes relataram buscar informações na leitura das placas expositivas, quando não tinham nenhuma informação sobre o animal que viam, nem que animal era. Nosso estudo confirma a relevância da leitura das placas como uma ferramenta eficaz para aquisição de informações e compartilhamento de conhecimento entre os membros familiares, já que eles as utilizaram para identificar ou rotular espécies, confirmar dados, complementar conhecimentos ou satisfazer a curiosidade.

Além disso, em nosso estudo, o conjunto recinto/animal vivo/placa dito como “a voz do zoológico” (Patrick, Tunnicliffe, 2013) foi uma tríade essencial que enriqueceu o conhecimento dos visitantes e a experiência sensorial, serviu como ferramenta para aprofundar as discussões e consolidar o aprendizado durante a visita ao zoológico. Também, a qualidade das placas se revelou crucial, já que placas atrativas despertam mais interesse (Saraiva, 2017), corroborando nossas observações pela quantidade de leituras associadas às conversas sobre ciência e animais e pelas observações das fotografias ou ilustrações para identificar as espécies. Acreditamos que a combinação de imagens e informações ampliou o engajamento cognitivo compartilhado, assim como mencionado por Aragão (2014).

As conversas familiares durante a visita ao zoológico também destacaram a importância dos atributos físicos e estéticos dos animais, como morfologia, tamanho e coloração. Entre esses elementos, o tamanho corporal e a cor foram identificados como determinantes para a atratividade dos animais, corroborando o estudo de Small (2012), no qual essas características tornam uma espécie atraente para os humanos em zoológicos.

A observação de características antropomórficas em mamíferos, tais como pelos e rosto com expressões emocionais, como as consideradas carismática, divertida e fofa, e/ou exibir características semelhantes às humanas, também despertou encantamento e empatia entre os familiares, alinhando-se com as conclusões de Mocada et al. (2002) e Carr (2016). No nosso estudo, esse encantamento do público visitante ocorreu principalmente ao se deparar com mamíferos, como felinos e macacos, resultado semelhante ao encontrado em Skibins, Dunstan, Pahlow (2017). Espécies carismáticas apresentam grande apelo social e impulsionam ações de conservação, como apontado por Martin et al. (2014).

Além das espécies carismáticas, este estudo também destaca a presença de conversas sobre animais considerados menos atrativos, como insetos. Assim, como no estudo de Small (2012), que sugere que os insetos são geralmente vistos por humanos como pouco atraentes, com exceções notáveis de borboletas e abelhas, no nosso estudo, esses insetos foram destacados. Acreditamos que a dedicação de um espaço específico para explicar seu ciclo de vida e comportamento evidencia uma estratégia eficaz para estimular o interesse dos visitantes, além dessa posição de destaque poder ser um caminho de estímulo à conservação.

Na categoria biológico, as estratégias de destaque para o envolvimento cognitivo e raciocínio dos grupos familiares foram expressas por meio do interesse deles de fazer perguntas, formular hipóteses e usar evidências para comunicar seus pensamentos, fazer inferências e revisar teorias sobre as temáticas expostas, alinhando-se ao trabalho de diversos autores (Bell et al., 2009; Kisiel et al., 2012; Land-Zandstra, Hoefakker, Damsma, 2020).

Bell et al. (2009) descrevem que entender o raciocínio científico em locais não formais, que são espaços científico-culturais de aprendizagem distintos do sistema formal de ensino, permite-nos compreender como as famílias se envolvem na investigação científica e como raciocinam e articulam seu pensamento em conversas com outras pessoas (Leinhardt, Crowley, Knutson, 2002; Povis, Crowley, 2015). Por isso, nessa categoria, ressaltamos a importância da linguagem que foi utilizada nas conversas sobre ciência e animais, com menções a experiências anteriores e comparações que podem contribuir para o desenvolvimento do raciocínio científico durante a interação familiar.

Observamos, no entanto, que, ao conversar, os familiares também recorreram a um raciocínio mais intuitivo e explicativo, com conceitos incompletos ou alternativos, mas que podem ser considerados uma forma válida de expressar conhecimento e experiências cotidianas. Reconhecemos que esse raciocínio desempenha um papel importante no desenvolvimento conceitual, trazendo aos grupos familiares o conteúdo científico de forma mais simples e potencializando a expressão do seu raciocínio em ambientes não formais (Massarani et al., 2022). Além disso, a coexistência de diferentes tipos de raciocínio nas conversas familiares destaca a complexidade do processo de aprendizagem e sugere que essas abordagens podem servir como um caminho de transição conceitual.

O envolvimento em conversas explicativas pode ser, nesse sentido, uma forma importante pela qual os adultos promovem a aprendizagem biológica em ambientes não formais (Legare, Lombrozo, 2014; Geerdts, Walle, Lobue, 2015), especialmente porque suas explicações auxiliam no desenvolvimento do conhecimento científico das crianças (Fender, Crowley, 2007). Em nosso estudo, percebemos que não só os pais, mas todas as conversas familiares serviram como base para experienciar o conhecimento científico e compreendê-lo, já que é nesses ambientes cotidianos que os familiares coconstroem o pensamento científico (Crowley et al., 2001). Tais conversas continham informações biológicas, conceituais e/ou explicativas, e acreditamos que essas conversas, por si só, já são uma forma de potencializar o raciocínio científico e as experiências de aprendizagem.

A discussão sobre as formas de aprendizado, como as respostas reflexivas dos familiares após explicações, destaca a importância do processamento cognitivo adicional na construção de significado (Ballantyne, Packer, Falk, 2011). Questionamentos sobre temas como tráfico, conservação e extinção de espécies indicam uma busca ativa por informações causais, revelando um interesse genuíno em entender o mundo ao redor, ler e explicar os processos biológicos de forma mais simples, reformulando-os. Ainda sobre esse assunto, entendemos a necessidade de incluir o histórico do animal no zoológico, principalmente quanto ao estado de conservação (Skibins, Dunstan, Pahlow, 2017) e ao tráfico ilegal, como visto nas placas do QuitoZoo. Isso ressalta o papel dos zoológicos não apenas como locais de entretenimento, mas também como espaços educativos cruciais para a conscientização sobre a conservação da vida selvagem (Clayton, Fraser, Saunders, 2009).

Por fim, a relevância de outros temas específicos, como alimentação, cuidado parental e camuflagem ressalta a diversidade de tópicos abordados nas conversas biológicas nos zoológicos e, em específico, no nosso estudo. A análise das conversas reflete a busca por informações específicas, como a dieta alimentar (Fraser et al., 2009) e o cuidado parental (Carr, 2016), destacando a importância de tópicos práticos e tangíveis para o público.

Na categoria conexão, destaca-se a importância das comparações e associações feitas pelos grupos familiares, sendo essa uma possível forma de aproximação dos animais e objetos expostos e, consequentemente, uma oportunidade de o público se envolver cognitivamente com eles, como apontado também por Namy e Gentner (2002). Embora as conversas possam ter sido abordadas superficialmente, com associações incompletas e concepções alternativas, reconhecemos sua relevância no desenvolvimento de conceitos e nas experiências de aprendizagem dos familiares, que se envolveram com diferentes perguntas e previsões e analisaram evidências no zoológico, o que reconhecemos como formas potenciais para expressar seus raciocínios, assim como no estudo museal de Massarani et al. (2022).

A conexão entre a experiência no zoológico e as vivências prévias, como situações cotidianas e visitas passadas, demonstra a tentativa de se relacionar o conteúdo expositivo com os eventos vivenciados pelas famílias (Silva, Gomes, 2009; Soares, Vieira, Fonseca, 2013). Essa estratégia de aproximação torna o assunto mais compreensível para crianças e adolescentes (Leinhardt, Crowley, Knutson, 2002), contribuindo para a significância da experiência no zoológico.

A manifestação de respostas emocionais, durante a visita, como destacado por Rupp (1999), também é discutida como uma ferramenta fundamental para otimizar as conexões entre o ambiente expositivo, a vida pessoal e as experiências anteriores dos familiares. As reações emocionais do nosso estudo foram frequentes e geralmente expressas em frases curtas, sendo classificadas como positivas e negativas. De qualquer modo, desempenharam um papel fundamental no envolvimento inicial das famílias com a exposição e, mesmo ocorrendo de forma pontual, podem potencializar a compreensão e a transferência de conhecimento em ambientes como os zoológicos (Caine, Caine, 1994; NRC, 2009).

As respostas emocionais positivas, como demonstrações de afeto pelos animais vivos, em diversos momentos da visita, evidenciam uma forte conexão entre os visitantes, os animais e a natureza (Clayton, Fraser, Burgess, 2011), como no Ex. 22 (G6) “A1: Ah, que coisinha. C1: Ah, meu amor, meu amor!” e Ex. 15 (G8) “C1: Eu gostaria de fazer carinho neles.”. Essa conexão pode ser um fator-chave na motivação para a aprendizagem de livre escolha e para as práticas pró-ambientais (Luebke et al., 2016), ao proporcionar espaços de afirmação para quem valoriza e se preocupa com os animais e ao promover uma experiência mais rica e significativa que desencadeia emoções.

Também ocorreu a identificação de respostas emocionais negativas (medo e nojo), em relação a certos animais, o que destaca a complexidade das emoções experimentadas pelos familiares, durante a visita ao zoológico. Apesar disso, essas emoções podem coexistir com uma curiosidade fascinante, conforme Clayton, Fraser e Saunders (2009) e, paradoxalmente, incentivar o interesse e a observação atenta desses animais (Rappolt-Schlichtmann, 2017).

As diversas reações emocionais dos grupos familiares demonstram a riqueza e a complexidade das experiências vivenciadas nos zoológicos, sendo a própria visita ao zoológico uma experiência que desperta o interesse em aprender mais sobre os animais (Luebke et al., 2016). A capacidade dos zoológicos de potencializar essas emoções e incentivar a discussão sobre elas pode ser essencial no suporte dos visitantes em iniciativas de conservação sobre a vida selvagem (Clayton, Fraser, Saunders, 2009).

## Considerações finais

Nosso objetivo foi aprofundar o conhecimento sobre a experiência familiar em zoológicos e entender como os visitantes constroem estratégias e interpretam o significado situacional da visita, analisando suas conversas, interações e reações emocionais. Nesse sentido, a visita familiar ao QuitoZoo pode ser caracterizada como uma vivência significativa na dimensão educativa e recreativa em um espaço não formal, associada a um momento de aprendizado em família, que gerou interesse, conhecimento e curiosidade.

As conversas que prevaleceram neste estudo estavam relacionadas à ciência e aos animais. Na categoria perceptivo, estratégias simples como manter a atenção compartilhada, identificar/rotular animais, promover diálogos específicos sobre eles, apontar e gesticular mostraram-se eficazes para apoiar a aprendizagem dos familiares, principalmente em ambientes visualmente atrativos e propensos à distração como os zoológicos.

Para embasar as conversas realizadas, a leitura das placas informativas emergiu como uma prática significativa, ao identificar espécies, confirmar informações e complementar conhecimentos. A qualidade das placas e sua atratividade com imagens contribuíram para o engajamento cognitivo compartilhado. Também se mostrou relevante o histórico dos animais, já que, muitas vezes, eles são resgatados do tráfico ilegal. Essa inclusão enriquece a experiência dos visitantes, promovendo uma conexão mais profunda com a missão do zoológico.

Na categoria biológico, a análise das conversas trouxe evidências de que o QuitoZoo pode funcionar como um local de aprendizado, sendo significativo na construção do pensamento e raciocínio científico com perguntas, hipóteses e uso de evidências. Os familiares também recorreram a um raciocínio mais intuitivo e explicativo, provavelmente como uma forma de expressar conhecimento e experiências cotidianas.

A coexistência de diversos tipos de raciocínio pode esboçar um caminho de transição conceitual. O envolvimento em conversas explicativas e em respostas reflexivas pode auxiliar na coconstrução do pensamento científico, enriquecendo o conhecimento biológico e conceitual dos familiares.

A análise da categoria conexão revelou a importância das comparações e associações feitas pelos familiares como uma forma de aproximação dos animais e objetos expostos, o que também pode proporcionar uma base para o envolvimento cognitivo com o ambiente do zoológico. Essa estratégia de interação cognitiva contribui para uma relação ativa dos familiares, que exploram perguntas, previsões e analisam evidências nesses ambientes.

A coexistência de emoções positivas e negativas ressalta a capacidade dos zoológicos em ampliar a conexão entre humanos e animais, podendo funcionar como um fator-chave na aprendizagem e na construção de significado. Também podem servir como catalisadores para práticas pró-ambientais em educação ambiental e na conservação das espécies.

Nossos resultados evidenciam o impacto da comunicação e divulgação científica em ambientes não formais. Isso pode servir como subsídio para profissionais de zoológicos na criação de estratégias que estimulem a participação e o debate dos visitantes sobre temas científicos.
